# Hydrostatic pressure effects on the static magnetism in Eu(Fe_0.925_Co_0.075_)_2_As_2_

**DOI:** 10.1038/s41598-017-03762-1

**Published:** 2017-06-14

**Authors:** W. T. Jin, J.-P. Sun, G. Z. Ye, Y. Xiao, Y. Su, K. Schmalzl, S. Nandi, Z. Bukowski, Z. Guguchia, E. Feng, Z. Fu, J.-G. Cheng

**Affiliations:** 1 Jülich Centre for Neutron Science JCNS at Heinz Maier-Leibnitz Zentrum (MLZ), Forschungszentrum Jülich GmbH, Lichtenbergstraße 1, D-85747 Garching, Germany; 2 Beijing National Laboratory for Condensed Matter Physics and Institute of Physics, Chinese Academy of Sciences, Beijing, 100190 China; 3 University of Chinese Academy of Sciences, Beijing, 100049 China; 4 Jülich Centre for Neutron Science JCNS and Peter Grünberg Institut PGI, JARA-FIT, Forschungszentrum Jülich GmbH, D-52425 Jülich, Germany; 5 Jülich Centre for Neutron Science JCNS at Institut Laue-Langevin (ILL), Forschungszentrum Jülich GmbH, Boite Postale 156, 38042 Grenoble Cedex 9, France; 6 Department of Physics, Indian Institute of Technology, Kanpur, 208016 India; 7 Institute of Low Temperature and Structure Research, Polish Academy of Sciences, 50-422 Wroclaw, Poland; 8 Department of Physics, Columbia University, New York, NY 10027 USA

## Abstract

EuFe_2_As_2_-based iron pnictides are quite interesting compounds, due to the two magnetic sublattices in them and the tunability to superconductors by chemical doping or application of external pressure. The effects of hydrostatic pressure on the static magnetism in Eu(Fe_0.925_Co_0.075_)_2_As_2_ are investigated by complementary electrical resistivity, ac magnetic susceptibility and single-crystal neutron diffraction measurements. A specific pressure-temperature (P-T) phase diagram of Eu(Fe_0.925_Co_0.075_)_2_As_2_ is established. The structural phase transition, as well as the spin-density-wave order of Fe sublattice, is suppressed gradually with increasing pressure and disappears completely above 2.0 GPa. In contrast, the magnetic order of Eu sublattice persists over the whole investigated pressure range up to 14 GPa, yet displaying a non-monotonic variation with pressure. With the increase of the hydrostatic pressure, the magnetic state of Eu evolves from the canted antiferromagnetic structure in the ground state, via a pure ferromagnetic structure under the intermediate pressure, finally to an “unconfirmed” antiferromagnetic structure under the high pressure. The strong ferromagnetism of Eu coexists with the pressure-induced superconductivity around 2 GPa. Comparisons between the P-T phase diagrams of Eu(Fe_0.925_Co_0.075_)_2_As_2_ and the parent compound EuFe_2_As_2_ were also made.

## Introduction

The discovery of Fe-based superconductors^1^ has provided new platforms to study the intriguing interplay between superconductivity (SC) and magnetism. SC in these novel materials was found to be in close proximity to the magnetism, as it emerges when the long-range antiferromagnetic (AFM) order in the parent compounds gets well suppressed by means of chemical doping or the application of external pressure^2^, and the spin fluctuations are believed to be responsible for the unconventional SC in them^3, 4^.

Among various classes of Fe-based superconductors, the EuFe_2_As_2_-based compounds (Eu-122) have drawn tremendous attention, as they contain two magnetic sublattices and show strong spin-charge-lattice coupling^5, 6^. In a purely ionic picture, the *S*-state (orbital moment *L* = 0) Eu^2+^ rare-earth ion has a 4*f* ^7^ electronic configuration and a total electron spin *S* = 7/2, corresponding to a theoretical effective magnetic moment of 7.94 *μ*_*B*_^7^. EuFe_2_As_2_ undergoes a structural phase transition from a tetragonal to an orthorhombic phase at 190 K, accompanied by a spin-density-wave (SDW) order of the itinerant Fe moments. In addition, the localized Eu^2+^ spins order below 19 K in the A-type AFM structure (ferromagnetic layers stacked antiferromagnetically along the *c* axis)^8–10^. The undoped parent compound EuFe_2_As_2_ can be tuned into a superconductor by chemical substitutions into the Eu-^11^, Fe-^12–14^, or As-site^15^, respectively. The SC can also be realized by the application of external physical pressure in undoped EuFe_2_As_2_ with the superconducting transition temperature *T*_*sc*_ ~ 30 K in a narrow range of 2.5–3.0 GPa^16–18^.

Recently, considerable experimental efforts have been devoted to understand how the magnetism in both sublattices develops with different chemical doping^12, 19–31^. It is well established that the SDW transition of the Fe sublattice gets gradually suppressed with increasing doping level in hole-doped Eu_1−*x*_K_*x*_Fe_2_As_2_^19^ and Eu_1−*x*_Na_*x*_Fe_2_As_2_^32^, in electron-doped Eu(Fe_1−*x*_Co_*x*_)_2_As_2_^21, 24^, Eu(Fe_1−*x*_Ir_*x*_)_2_As_2_^14, 33^, and Eu_1−*x*_La_*x*_Fe_2_As_2_^20^, as well as in isovalent-substituted Eu(Fe_1−*x*_Ru_*x*_)_2_As_2_^30^ and EuFe_2_(As_1−*x*_P_*x*_)_2_^25, 26^, while the magnetic order of the Eu sublattice persists over the whole doping region. The magnetic ground state of the Eu^2+^ moments displays a systematic change with increasing doping concentration, from the A-type AFM structure (with the spins lying in the *ab* planes) at low doping levels to the pure ferromagnetic structure (with the spins aligning along the *c* axis) at high doping levels^24^. Interestingly, the strong ferromagnetic (FM) order of the localized Eu^2+^ spins, with a huge moment close to 7 *μ*_*B*_ per Eu, was confirmed to be compatible with the SC^13–15, 33–37^.

Nevertheless, to the best of our knowledge, only a limited number of studies about the pressure effects on the Eu magnetism in the Eu-122 compounds exist. For the undoped parent compound EuFe_2_As_2_, the high-pressure ac magnetic susceptibility measurement using the piston-cylinder cell suggests that the magnetic ground state of the Eu^2+^ moments is still an AFM order in the pressure-induced superconducting phase (with the maximum applied pressure *P* ~ 2.8 GPa)^17^, similar to that under the ambient pressure. Further measurements using a cubic anvil cell indicate that the AFM order of the Eu^2+^ moments persists up to an applied pressure *P* ~ 6 GPa, above which it changes to the FM order, as confirmed by high-pressure x-ray magnetic circular dichroism (XMCD) experiments^18^. The SDW transition of Fe gets completely suppressed at the critical pressure *P*_*C*_ where the SC emerges. In addition, complementary high-pressure muon-spin rotation (*μ*SR) and magnetization measurements were performed on non-superconducting isovalent-substituted EuFe_2_(As_0.88_P_0.12_)_2_, in which the Eu^2+^ spins were found to order in the canted AFM (C-AFM) structure in the ground state^28^. Possible superconducting phase (“X” phase as referred in ref. 28) was realized in EuFe_2_(As_0.88_P_0.12_)_2_ within a very narrow pressure range of 0.36–0.5 GPa, coexisting with the magnetic order of both the Eu and Fe moments. However, the magnetic structure of Eu in the so-called “X” phase can not be unambiguously determined there.

In order to conclude how the magnetism in both sublattices develop with the external pressure and to clarify the nature of the magnetic state in possible pressure-induced superconducting phase, we have carried out complementary experiments including the electrical resistivity, ac magnetic susceptibility and neutron diffraction measurements on the Eu(Fe_1−*x*_Co_*x*_)_2_As_2_ (*x* = 0.075) single crystal under hydrostatic pressure. There are two reasons of choosing Eu(Fe_0.925_Co_0.075_)_2_As_2_ for the high-pressure studies. Firstly, according to the established *T* − *x* phase diagram of electron-doped Eu(Fe_1−*x*_Co_*x*_)_2_As_2_^24^, the sample with *x* = 0.075 is close to the superconducting dome. The superconducting phase might be reachable by applying moderate hydrostatic pressure. Secondly, the magnetic ground-state of the Eu sublattice in Eu(Fe_0.925_Co_0.075_)_2_As_2_ has been determined to be a canted-AFM structure^24^. The Eu^2+^ spins are canted out of the *ab* planes with an angle of 23.8(6)°, giving rise to a net ferromagnetic moment component along the *c* axis. It is thus very interesting to investigate how this intermediate magnetic structure in the *T* − *x* phase diagram evolves with hydrostatic pressure, and to conclude what type of magnetic order of Eu can coexist with the pressure-induced superconducting phase.

## Results

**Figure 1:**
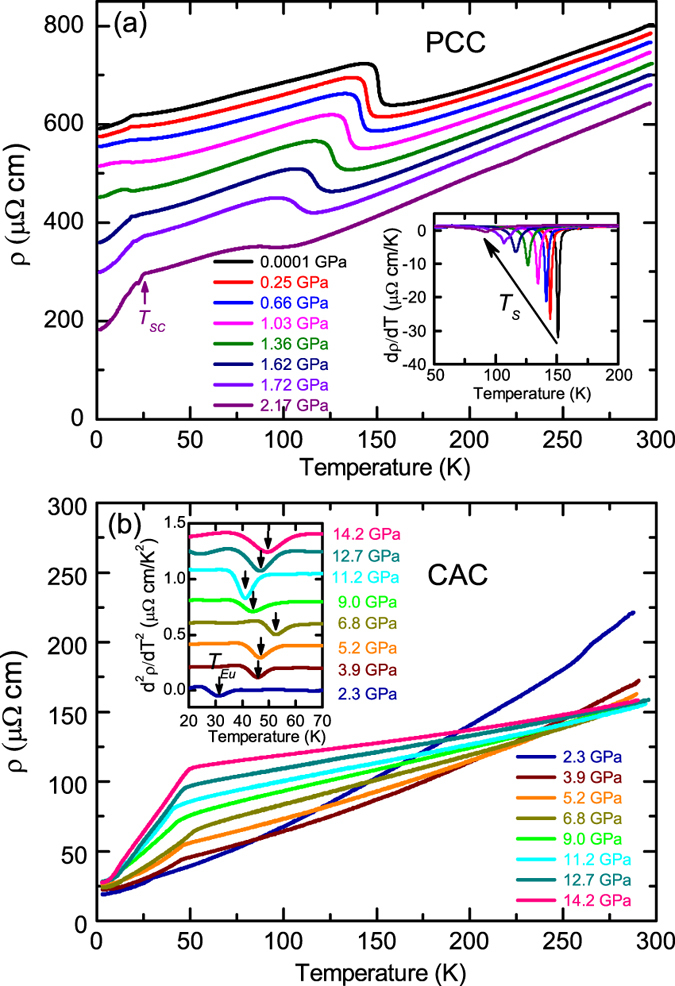
Temperature dependencies of the electrical resistivity (*ρ*) of the Eu(Fe_0.925_Co_0.075_)_2_As_2_ single crystals measured under high pressures with the piston-cylinder cell (**a**) and the cubic anvil cell (**b**), respectively.

Figure 1 shows the temperature dependencies of the electrical resistivity, *ρ*(*T*), of the Eu(Fe_0.925_Co_0.075_)_2_As_2_ single crystal measured with the piston-cylinder cell (PCC) (a) and the cubic anvil cell (CAC) (b), respectively. At ambient pressure, an upturn in *ρ*(*T*) appears at *T*_*S*_ ~ 152 K, corresponding to the structural phase transition, as confirmed by neutron diffraction presented below. Here *T*_*S*_ is defined as the minimum in the first derivative of *ρ*(*T*), *dρ*(*T*)/*dT*. In addition, *ρ*(*T*) shows another kink at *T*_*Eu*_= 17 K, due to the magnetic order of the Eu^2+^ moments. The ambient-pressure *ρ*(*T*) curve of Eu(Fe_0.925_Co_0.075_)_2_As_2_ was measured on a small strip cut from the crystal and does not show a drastic drop below 11.4 K, indicating the filamentary nature of the superconducting-like behavior observed on a much larger crystal (Fig. 1 in ref. 24). With increasing pressure, *T*_*S*_ shifts gradually to the lower temperature, as shown in the inset of Fig. 1(a). Above 2.3 GPa, no upturn in *ρ*(*T*) can be observed anymore (Fig. 1(b)), indicating that the structural and SDW transitions get completely suppressed at this pressure. However, *T*_*Eu*_, the magnetic transition temperature of Eu, seems quite insensitive to the applied pressure and stays lower than 20 K for *P* ≤ 2 GPa. As shown in the inset of Fig. 1(b), with further increasing pressure, *T*_*Eu*_ starts to change significantly, as revealed by the non-monotonous change of the minimum in the second derivative of *ρ*(*T*), *d* ^2^ *ρ*(*T*)/*dT* ^2^. After reaching a maximum value of ~52 K at 6.8 GPa, *T*_*Eu*_ decreases slightly with increasing pressure, to ~41 K at 11.2 GPa. Interestingly, *T*_*Eu*_ reverses to increase again when more pressure is applied. At the maximum applied pressure of 14.2 GPa, *T*_*Eu*_ reaches another maximum around 49 K. It is worth noting that at 2.17 GPa, *ρ*(*T*) shows a sharp drop at *T*_*SC*_ ~ 25 K, suggesting the appearance of the pressure-induced SC as reported previously in the parent compound^16, 18^. The superconducting nature at 2.17 GPa is also reflected in the ac magnetic susceptibility data presented below. However, the transition to a zero-resistivity state is hindered by the magnetic order of Eu, as shown by another anomaly in *ρ*(*T*) around 21 K. The pressure-induced reentrant resistivity below the superconducting transition here resembles that observed at ambient pressure in the Eu(Fe_0.89_Co_0.11_)_2_As_2_ single crystals grown from self-flux method^12^, ascribing to the competition between the SC and the Eu magnetic order.

**Figure 2:**
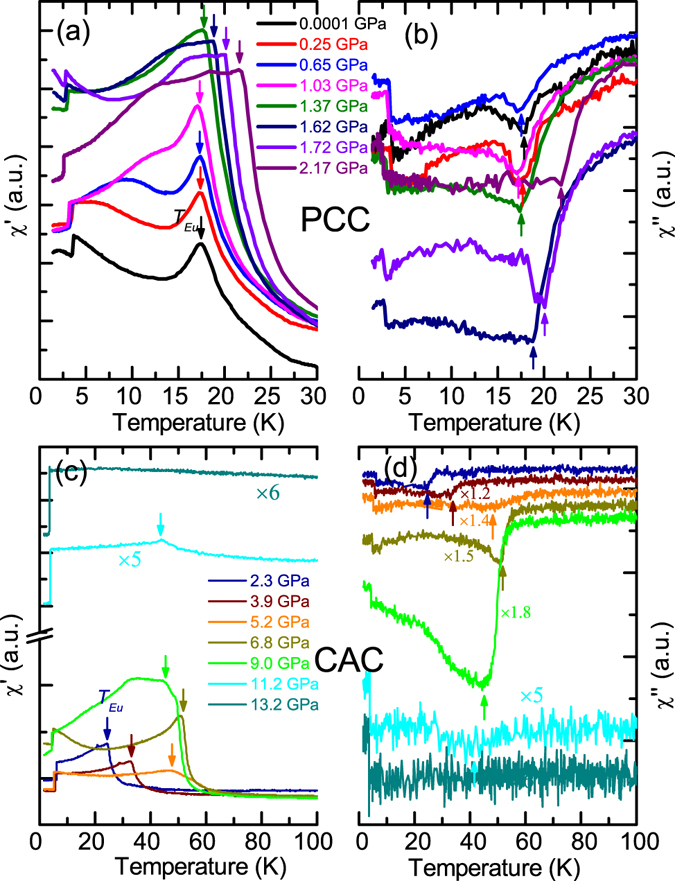
Temperature dependencies of the real and imaginary part of the ac magnetic susceptibility, *χ*′ and *χ*″, of the Eu(Fe_0.925_Co_0.075_)_2_As_2_ single crystals measured under high pressures with the piston-cylinder cell (**a**,**b**) and the cubic anvil cell (**c**,**d**), respectively.

The ac magnetic susceptibility of the Eu(Fe_0.925_Co_0.075_)_2_As_2_ single crystal measured under high pressure with the PCC and the CAC is shown in Fig. 2(a,b), respectively. At ambient pressure, a peak at *T*_*Eu*_ = 17 K in *χ*′, the real part of the ac magnetic susceptibility, indicates the antiferromagnetic order of the Eu^2+^ spins. As determined from ambient-pressure neutron diffraction, the magnetic ground state of the Eu^2+^ moments is a canted AFM structure with a net FM moment component along the *c* axis^24^. With increasing pressure, *T*_*Eu*_ shifted slightly to a lower temperature (with *dT*_*Eu*_/*dP* ~ −0.3 K/GPa) for *P* ≤ 1.03 GPa, as shown in Fig. 2(a), suggesting that the interlayer AFM coupling weakens under pressure. At 1.37 GPa, the antiferromagnetic peak almost gets smeared out and instead a pronounced kink is observed at *T*_*Eu*_ = 18 K. The kink temperature shifts continuously to a higher temperature with further increasing pressure, reaching a maximum value of ~52 K at 6.8 GPa (Fig. 2(c)). The distinct tendencies of the evolution of *T*_*Eu*_ with increasing pressure for *P* ≤ 1.03 GPa and 1.37 GPa ≤ *P* ≤ 6.8 GPa suggests that the magnetic structures of the Eu^2+^ spins might be different in the two pressure regions. With further increasing pressure (*P* ≥ 6.8 GPa), *T*_*Eu*_ decreases again, as shown in Fig. 2(c), to ~43 K at 11.2 GPa. The pressure dependence of *T*_*Eu*_ for Eu(Fe_0.925_Co_0.075_)_2_As_2_ obtained from the ac magnetic susceptibility measurement is well consistent with that extracted from the resistivity measurement. The anomaly at *T*_*Eu*_ can be hardly resolved in *χ*′ for *P* ≥ 13.2 GPa, implying that the Eu^2+^ moments probably order antiferromagnetically again. Furthermore, *χ*′ shows additional features for the pressures around 2.17 GPa. Compared with other pressures, the *χ*′(*T*) curves at 2.17 GPa exhibits some diamagnetic response associated with superconductivity. As shown in Fig. 2(a), at *P* = 2.17 GPa, the slope of the *χ*′(*T*) curve shows a pronounced downward bending around 12 K, which is most likely the result of the competition between the magnetism of Eu and the pressure-induced superconductivity, as hinted by the resistivity data at this pressure value. A similar diamagnetic response in *χ*′ was reported as the evidence for the pressure-induced superconductivity at 2.5 GPa for the parent compound EuFe_2_As_2_^18^. Unfortunately, in our case, the cubic anvil cell can not generate a pressure close enough to 2.17 GPa to validate the zero-resistance state in our sample (note that the superconducting dome is extremely narrow as shown in Fig. 7 of ref. 18).

**Figure 3:**
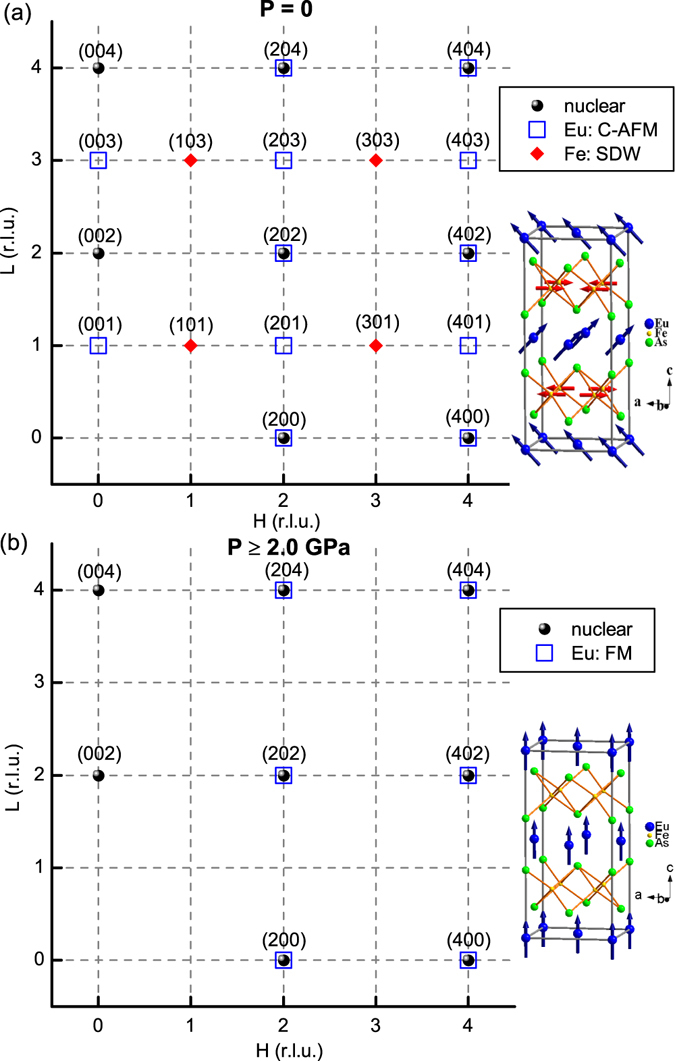
The magnetic structures of Eu(Fe_0.925_Co_0.075_)_2_As_2_ at the base temperature for *P* = 0 (**a**) and *P* ≥ 2.0 GPa (**b**), respectively. The expected nuclear peaks (black spheres) and magnetic reflections from Eu (blue squares) and Fe (red diamonds) in the (H 0 L) reciprocal plane are illustrated. At *P* = 0 (**a**), the Eu sublattice orders in the canted AFM structure and the Fe sublattice shows a SDW order. For *P* ≥ 2.0 GPa (**b**), the Eu sublattice shows a pure FM order and the antiferromagnetism in the Fe sublattice is completely suppressed.

**Figure 4:**
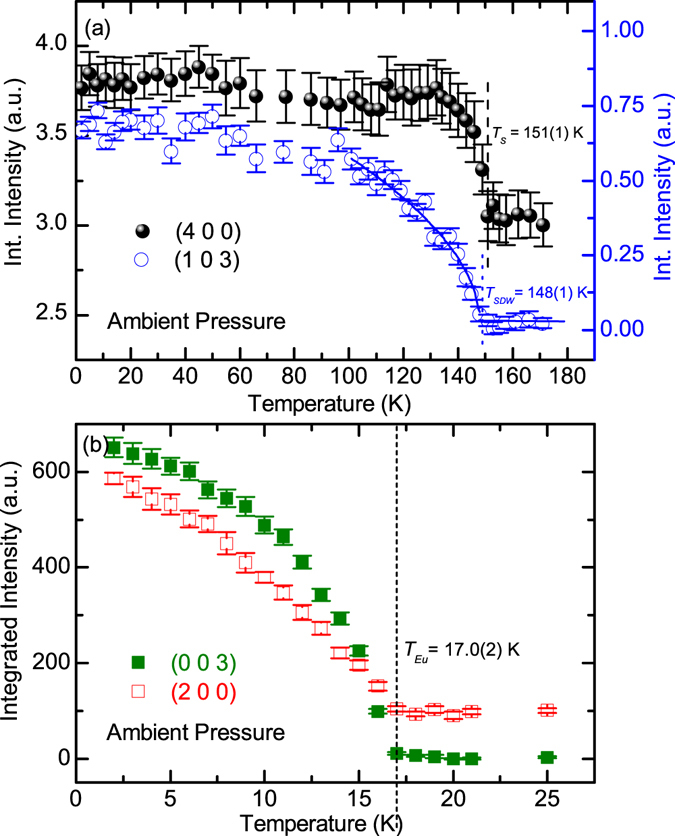
Temperature dependencies of the integrated intensity of the (4 0 0) and (1 0 3) reflections (**a**), and the (0 0 3) and (2 0 0) reflections (**b**), respectively, measured by neutron diffraction on the Eu(Fe_0.925_Co_0.075_)_2_As_2_ single crystal at ambient pressure. The dash line and dot line in (**a**) mark the structural phase transition and the SDW transition of Fe, respectively. The short dash line in (**b**) marks the magnetic transition associated with the canted AFM order of the Eu^2+^ spins.

In order to confirm the nature of the anomalies revealed by the macroscopic measurements, the neutron diffraction measurements were performed on the Eu(Fe_0.925_Co_0.075_)_2_As_2_ single crystal at ambient pressure and under high pressure, respectively. The magnetic structures of Eu(Fe_0.925_Co_0.075_)_2_As_2_ at the base temperature for *P* = 0 and *P* ≥ 2.0 GPa were determined and shown in Fig. 3(a,b), respectively, in which the expected nuclear and magnetic reflections in the (H 0 L) reciprocal plane were illustrated. Figure 4(a) shows the ambient-pressure temperature dependencies of the integrated intensity of the (4 0 0) and (1 0 3) peaks, one strong nuclear reflection of the orthorhombic phase and one magnetic reflection due to the SDW order of the itinerant Fe moments, respectively. The rapid increase of the intensity of the nulear (4 0 0) peak below *T*_*S*_ = 151(1) K indicates the structural phase transition from a tetragonal to an orthorhombic phase, as the emergence of the orthorhombic domains has a strong impact on the extinction conditions of the nuclear Bragg reflections. The transition temperature determined here is well consistent with that determined from the resistivity measurement. In addition, fitting to the intensity of the (1 0 3) reflection for the temperature close to the transition yields the onset temperature of the SDW order of Fe, *T*_*SDW*_ = 148(1) K. Compared with the parent compound EuFe_2_As_2_, both transitions are significantly suppressed by 7.5% Co doping. The size of the Fe^2+^ moment is estimated to be 0.63(4) *μ*_*B*_. Furthermore, at ambient pressure, the magnetic ground state of Eu was determined to be a canted AFM structure with a net FM moment component along the *c* axis, as reported in ref. 24. The Eu^2+^ spins were found to be canted with an angle of 23.8(6)° out of the *ab* plane with the moment size of 6.22(3) *μ*_*B*_, as shown in Fig. 3(a). The magnetic ordering temperature of Eu was determined to be 17.0(2) K according to the temperature dependencies of both the (2 0 0) and (0 0 3) magnetic peaks, as shown in Fig. 4(b).

**Figure 5:**
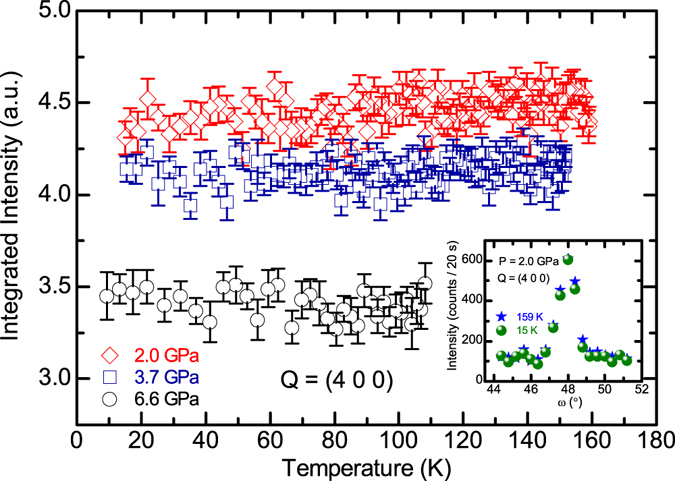
Temperature dependencies of the integrated intensity of the (4 0 0) nuclear reflection of the Eu(Fe_0.925_Co_0.075_)_2_As_2_ single crystal under the high pressure at 2.0 GPa, 3.7 GPa and 6.6 GPa, respectively. The rocking scans of the (4 0 0) peak at 159 K and 15 K under the pressure at 2.0 GPa are shown in the inset.

Figure 5 shows the temperature dependencies of the integrated intensity of the (4 0 0) nuclear peak measured under the high pressure at 2.0 GPa, 3.7 GPa and 6.6 GPa, respectively. Different from a rapid increase below *T*_*S*_ = 151(1) K at ambient pressure (see Fig. 4(a)), the intensity of (4 0 0) remains almost constant for the temperature range from 15 K to 160 K when a pressure of 2.0 GPa is applied, as shown in the inset of Fig. 5, indicating the complete suppression of the structural phase transition in Eu(Fe_0.925_Co_0.075_)_2_As_2_ under the pressure larger than 2.0 GPa (*P* ≥ 2.0 GPa). This is slightly inconsistent with the result from the resistivity measurements, since an upturn can still be resolved around 92 K in *ρ*(*T*) at 2.17 GPa. The discrepancy between two probes might be due to the difference in the hydrostaticity of the pressure generated by the piston-cylinder cell and the Paris-Edinburgh cell. As presented above, the signature of superconductivity is exhibited in the macroscopic measurements for the pressure close to 2.17 GPa. Therefore, the complete suppression of the structural phase transition at 2.0 GPa is in line with the expectation that the superconductivity emerges in close proximity to the criticality where the structural distortion, as well as the following or accompanying SDW order of Fe, disappears.

**Figure 6:**
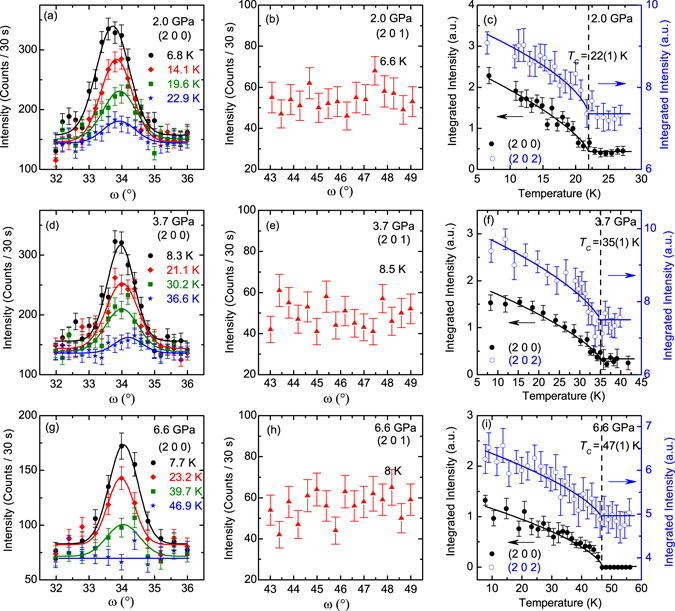
The summary of the neutron diffraction data on the Eu(Fe_0.925_Co_0.075_)_2_As_2_ single crystal measured under the high pressure at 2.0 GPa (**a**–**c**), 3.7 GPa (**d**–**f**) and 6.6 GPa (**g**–**i**), respectively. The rocking scans of the (2 0 0) and (2 0 1) reflections at different temperatures measured at 2.0 GPa, 3.7 GPa, and 6.6 GPa are shown in (**a**,**b**,**d**,**e**,**g**,**h**), respectively. The solid curves represent the fits using the Gaussian profiles. The integrated intensity of the (2 0 0) and (2 0 2) peaks under different pressure values are plotted as functions of the temperature in (**c**,**f**,**i**), respectively. The vertical dashed lines mark the ferromagnetic transition temperatures of Eu at different *pressures* and the solid lines are guides to the eye.

The effect of hydrostatic pressure on the magnetic reflections arising from the magnetic order of Eu is summarized in Fig. 6. As shown in Fig. 6(a,d,g), the intensity of the (2 0 0) peak always weakens significantly with increasing temperature, indicating a strong ferromagnetic contribution on this reflection, at 2.0, 3.7 and 6.6 GPa, respectively. On the other hand, the (2 0 1) reflection observed at ambient pressure due to the antiferromagnetic interlayer coupling of the Eu^2+^ spins disappears upon the application of the pressure larger than 2.0 GPa (Fig. 6(b,e,h)), suggesting a pure ferromagnetic order of the Eu^2+^ moments along the *c* axis at the applied pressure values. The temperature dependencies of the integrated intensity of both the (2 0 0) and (2 0 2) reflections as plotted in Fig. 6(c,f,i) allow us to determine the ferromagnetic transition temperature (*T*_*C*_) as 22(1), 35(1) and 47(1) K, for 2.0, 3.7 and 6.6 GPa, respectively. The observation here that *T*_*C*_ shifts to a higher temperature with increasing pressure is well consistent with the results from both the resistivity and the ac magnetic susceptibility measurements within the same pressure region. It is worth noting that the net ferromagnetic contribution on the nuclear scattering part of the (2, 0, 2) reflection at the lowest temperature is estimated to be around 24 % for all the three pressure values, further corroborating the ferromagnetic alignment of the Eu^2+^ moments completely along the *c* axis at 2.0, 3.7 and 6.6 GPa^24, 34^.

## Discussion

**Figure 7:**
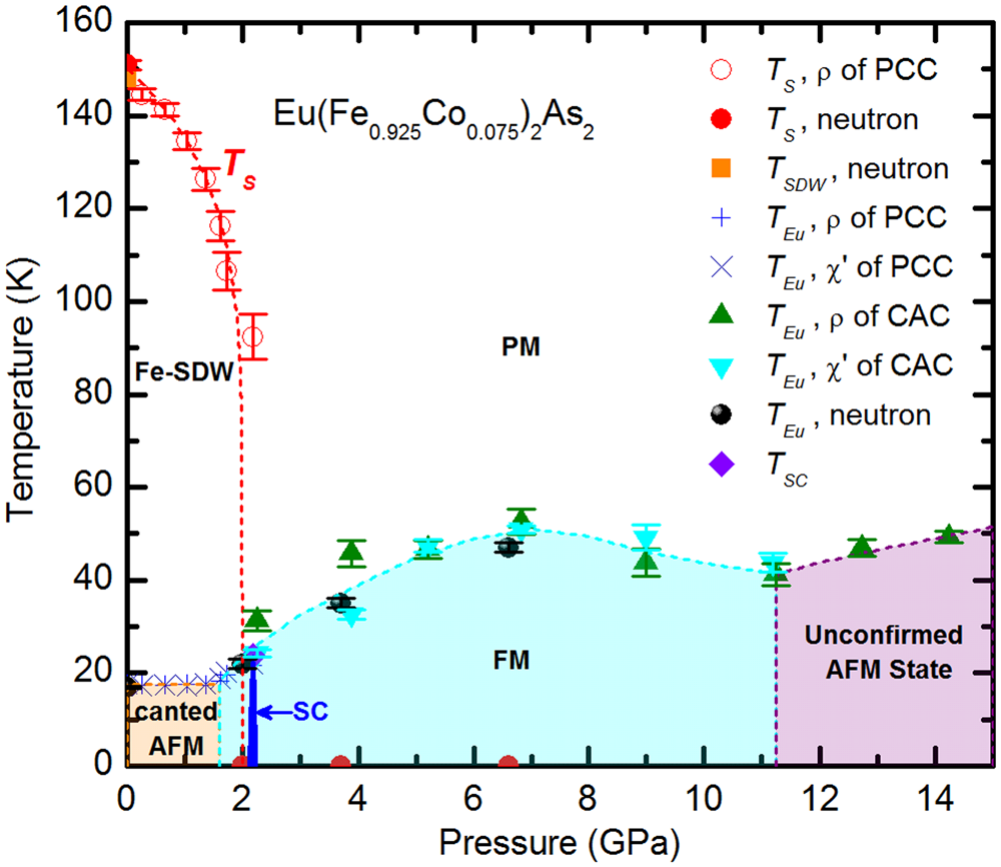
Pressure-temperature phase diagram of Eu(Fe_0.925_Co_0.075_)_2_As_2_ determined from the resistivity, ac magnetic susceptibility, and neutron diffraction measurements.

Combining the results from the resistivity, ac magnetic susceptibility and neutron diffraction measurements, a phase diagram describing how the static magnetism of Eu(Fe_0.925_Co_0.075_)_2_As_2_ develops upon the application of hydrostatic pressure is established. As shown in Fig. 7, the structural phase transition (as well as the SDW order of Fe) gets gradually suppressed with increasing pressure and disappears at a critical pressure *P*_*c*_ ~ 2.0 GPa, which is lower than *P*_*c*_ ~ 2.5–2.7 GPa for the parent compound EuFe_2_As_2_^18^, ascribing to additional electron-doping effect from Co. Compared with other compounds within the “122” family, the critical pressure of the EuFe_2_As_2_ system is found to be lower than that of BaFe_2_As_2_ (*P*_*c*_ ~ 4 GPa) and SrFe_2_As_2_ (*P*_*c*_ ~ 3.6–3.7 GPa) determined from resistivity measurements using the same Daphne Oil as the pressure medium^38, 39^, but higher than that of CaFe_2_As_2_ (*P*_*c*_ ~ 0.5 GPa) determined using the Silicone Oil as the pressure medium^40^.

The magnetic order of Eu persists over the whole range of the applied pressure up to 14 GPa, yet displaying a non-monotonic variation with pressure. Below 1.5 GPa, *T*_*Eu*_, the ordering temperature of the Eu^2+^ spins, stays almost constant, suggesting a canted antiferromagnetic structure with a net ferromagnetic moment component along the *c* direction, as observed at the ambient pressure. With further increasing pressure, *T*_*Eu*_ starts to increase, reaching a maximum value of ~50 K at 7 GPa. The magnetic structure of Eu in this pressure range is revealed by neutron diffraction to be a pure ferromagnetic order along the *c* axis. The role of the hydrostatic pressure in driving the Eu^2+^ moments to order ferromagnetically in Eu(Fe_0.925_Co_0.075_)_2_As_2_ seems quite similar to the effect of introducing more electrons by the means of further Co doping, as reflected by the ambient-pressure phase diagram of Eu(Fe_1−*x*_Co_*x*_)_2_As_2_^24^. Above 7 GPa, *T*_*Eu*_ declines slightly with increasing pressure to ~40 K at 11.2 GPa, indicating the weakening of the ferromagnetic coupling between the Eu^2+^ moments in this pressure range. The suppression of the FM state was also observed in the parent compound EuFe_2_As_2_ for *P* ≥ 8 GPa, which was explained as the result of the valance change from magnetic Eu^2+^ to nonmagnetic Eu^3+^ state as observed by x-ray absorption spectroscopy (XAS) under high pressure^18^. The pressure dependence of *T*_*Eu*_ in Eu(Fe_0.925_Co_0.075_)_2_As_2_ for *P* ≤ 11 GPa is quite similar to that in the parent compound EuFe_2_As_2_^18^, showing an almost constant value in the AFM or canted AFM region and a dome-like variation in the FM region. It was also found that the shifting of the kink below 50 K in the resistivity, *ρ*(*T*), of EuFe_2_As_2_ towards the lower temperature might be correlated with the pressure-induced transition to a collapsed tetragonal (CT) phase^41, 42^. However, our current neutron data does not allow a precise structural determination to validate if such a CT phase exists in Eu(Fe_0.925_Co_0.075_)_2_As_2_ for *P* ≥ 6.6 GPa.

Interestingly, as revealed by the resistivity measurements, *T*_*Eu*_(*P*) reverses to increase again for Eu(Fe_0.925_Co_0.075_)_2_As_2_ when more pressure is applied, which was not observed for the parent compound. As revealed by high-pressure XAS, the average valence state of Eu in both EuFe_2_As_2_ and EuCo_2_As_2_ increases with the applied pressure due to a part transition from Eu^2+^ to Eu^3+^. However, the mean valence in them gets stabilized around +2.3 at 10 GPa and +2.25 at 12.6 GPa, for EuFe_2_As_2_ and EuCo_2_As_2_, respectively^43, 44^. Therefore, the dip in *T*_*Eu*_(*P*) around 11 GPa in Fig. 7 is most likely due to combined effects of the pressure-induced valence change of Eu and the pressure-driven modification of the indirect Ruderman-Kittel-Kasuya-Yoshida (RKKY) exchange interaction in Eu(Fe_0.925_Co_0.075_)_2_As_2_. Since the RKKY exchange coupling depends strongly on the distance between interlayer Eu^2+^ moments, which is closely related to the applied hydrostatic pressure, it is expectable that the magnetic state of Eu as well as the ordering temperature, *T*_*Eu*_, will be tuned accordingly with increasing pressure. Unfortunately, due to the limitation of the Paris-Edinburgh pressure cell used in the neutron diffraction experiment, we can not achieve the pressure above 11 GPa so as to conclude about the nature of the magnetic state of Eu in this pressure region. The unobservable anomaly in the ac susceptibility data at 13.2 GPa as shown in Fig. 2(c) tends to support an antiferromagnetic order (either commensurate or incommensurate) of the Eu sublattice. Therefore, we refer it as an “unconfirmed AFM state” in Fig. 7.

In addition, as hinted by the macroscopic measurements, the signature of superconductivity emerges around 2.0 GPa. Therefore, the strong ferromagnetic order of Eu at 2.0 GPa is compatible with the pressure-induced superconductivity for Eu(Fe_0.925_Co_0.075_)_2_As_2_, resembling the well confirmed coexistence of Eu-FM and the doping-induced SC in several families of doped EuFe_2_As_2_^13–15, 24, 33–36^. Similar to that of the parent compound EuFe_2_As_2_^18^, the pressure-induced superconducting dome of Eu(Fe_0.925_Co_0.075_)_2_As_2_, is quite narrow. By comparison with BaFe_2_As_2_, SrFe_2_As_2_, and CaFe_2_As_2_^45, 46^, in which the superconducting domes in the P-T phase diagrams are much broader, it is clear that the magnetic order of Eu is unfavorable for the occurence of superconductivity in the EuFe_2_As_2_ system.

In conclusion, the effects of hydrostatic pressure on the static magnetism in Eu(Fe_0.925_Co_0.075_)_2_As_2_ are investigated by complementary electrical resistivity, ac magnetic susceptibility and single-crystal neutron diffraction measurements. A specific pressure-temperature phase diagram of Eu(Fe_0.925_Co_0.075_)_2_As_2_ is established. The structural phase transition, as well as the spin-density-wave order of Fe sublattice, is suppressed gradually with increasing pressure and disappears completely above 2.0 GPa. In contrast, the magnetic order of Eu sublattice persists over the whole investigated pressure range up to 14 GPa, yet displaying a non-monotonic variation with pressure. With the increase of the hydrostatic pressure, the magnetic state of Eu evolves from the canted antiferromagnetic structure in the ground state, via a pure ferromagnetic structure under the intermediate pressure, finally to an “unconfirmed” antiferromagnetic structure under the high pressure. The strong ferromagnetism of Eu coexists with the pressure-induced superconductivity around 2 GPa. Comparisons between the P-T phase diagrams of Eu(Fe_0.925_Co_0.075_)_2_As_2_ and the parent compound EuFe_2_As_2_ were also made.

## Methods

### Crystal growth

The single crystal of Eu(Fe_0.925_Co_0.075_)_2_As_2_ was grown out of the Sn flux^24^. The concentration of Co was determined by wavelength dispersive spectroscopy (WDS). The crystal is of a single phase, as all reflections in the neutron diffraction experiment were found to be well centered and indexed, and no reflections from possible impurity phases were observed. The ambient-pressure electrical resistivity measured on several strips cut from the same crystal show a similar behavior with an upturn in *ρ*(*T*) around the same temperature, *T*_*S*_ = 152(1) K, indicating good homogeneity of the crystal, as *T*_*S*_ strongly depends on the Co concentration in Eu(Fe_1−*x*_Co_*x*_)_2_As_2_^24^.

### Ambient- and high-pressure neutron diffraction measurements

Both the ambient-pressure and high-pressure neutron diffraction experiments were performed on the thermal-neutron two-axis diffractometer D23 at the Institut Laue Langevin (Grenoble, France). A Cu (2 0 0) monochromator was chosen to produce a monochromatic neutron beam with the wavelength of 1.279 Å. For the ambient-pressure measurement, a 76 mg platelike single crystal with dimensions ∼ 6 × 5 × 1 mm^3^ was mounted on a thin Al plate with a small amount of GE varnish, and put inside a standard orange cryostat. For the high-pressure measurement, a 10 mg rectangular strip with dimensions ∼ 4 × 1 × 1 mm^3^ was cut from the same piece of crystal, and put inside a TiZr gasket together with some lead powders as the pressure medium. The gasket was then mounted into the VX-5 type Paris-Edinburgh pressure cell^47^ loaded with He gas for low-temperature measurements in a 4 K dedicated cryostat. The pressure values were determined from the equation of state of lead^48^, based on the lattice parameters of lead measured by neutron diffraction at a certain temperature. For both experimental conditions, the crystals were oriented with the orthorhombic *b* axis (or *a* axis due to twinning) lying vertical, so that the (*H* 0 *L*) scattering plane can be accessible horizontally. (The orthorhombic notation is used throughout this paper for convenience).

### High-pressure resistivity and ac magnetic susceptibility measurements

High-pressure resistivity and ac magnetic susceptibility were measured in the Institute of Physics, Chinese Academy of Sciences, by using a self-clamped piston-cylinder cell (PCC) up to 2.2 GPa and a “Palm” cubic anvil cell (CAC) up to 14.2 GPa. The standard four-probe method was used for the resistivity measurements and the mutual induction method for the ac magnetic susceptibility measurements. The pressure inside the PCC was determined by monitoring the superconducting transition temperature of tin (Sn), which was placed together with the sample in the Teflon cell filled with the Daphne 7373 as the pressure transmitting medium (PTM). The pressure inside the CAC was calibrated at room temperature by observing the characteristic phase transitions of Bismuth (Bi). In this case, glycerol was used as the PTM.
